# Exploring the fungal community structure and assembly in different tissues of *Gymnadenia conopsea*

**DOI:** 10.3389/fmicb.2025.1640133

**Published:** 2025-10-10

**Authors:** Xiu Yin, Yazhou Lu, Hong Quan, Erhao Zhang, Zhongbin Wang

**Affiliations:** ^1^The Provincial and Ministerial Co-founded Collaborative Innovation Center for R&D in Xizang Characteristic Agricultural and Animal Husbandry Resources, Xizang Agriculture and Animal Husbandry University, Linzhi, China; ^2^Key Laboratory of Traditional zang's Medicine Resources Conservation and Utilization of Xizang Autonomous Region, Xizang Agriculture and Animal Husbandry University, Linzhi, China; ^3^Resources and Environment College, Xizang Agriculture and Animal Husbandry University, Linzhi, China

**Keywords:** *Gymnadenia conopsea*, endophytic fungi, community structure, network, assembly, tissue type

## Abstract

*Gymnadenia conopsea* has high economic value and can be used as a medicinal and ornamental plant. Owing to its low natural reproduction rate and overexploitation, the risk of extinction of this plant is gradually increasing. Endophytic fungi play crucial roles in host growth and development; however, the characteristics of the endophytic fungal community in various tissues of *G. conopsea* have not been fully characterized. Illumina MiSeq high-throughput sequencing technology was employed to sequence the fungal ITS (A non-coding DNA sequence located between the 18S rRNA gene and the 5.8S rRNA gene within the ribosomal RNA gene cluster) region, thereby characterizing the community structure and assembly processes of endophytic fungi in roots, stems, leaves, and fruits. A total of 7,371 OTUs were obtained from all the samples and were dominated by Ascomycota and Basidiomycota. The richness indices of various tissues were significantly different, whereas the diversity indices were not significantly different. The composition of the dominant genera differed; overall, the compositions of the endophytic fungal communities were similar among the leaf, stem, and fruit tissues. The relative abundances of *Ceratobasidium, Cadophora*, and *Mortierella* were significantly higher in root tissues than in other tissues, *G. conopsea* roots should be prioritized as the material for isolating growth-promoting endophytic fungi. Cooccurrence network analysis revealed that endophytic fungi in different tissues presented typical modular structures and the network was mainly positive. The assembly processes in different tissues were affected mainly by deterministic factors. The proportions of pathotrophs, saprotrophs, and symbiotrophs were different among various tissues, and the proportion of pathotrophs was greater than those of saprotrophs and symbiotrophs. In conclusion, tissue type affects the composition of endophytic fungi. Furthermore, by dissecting the composition and functions of the fungal community associated with *G. conopsea*, this study aims to provide a reference for exploring the interaction mechanisms between endophytic fungi and *G. conopsea*.

## 1 Introduction

*Gymnadenia conopsea* (L.) R. Br, which is widely distributed in Asia and Europe, is a perennial and terrestrial herb of the Orchidaceae family (Lin et al., 2020; Shi et al., 2023). In Xizang of China, *G. conopsea* grows mainly in forests, grasslands, and waterlogged meadows at altitudes of 1,265–4,700 m (Shang et al., 2017). *G*. *conopsea* is a valuable traditional medicine and edible plant, and its tubers have multiple benefits, including antifatigue (Zhao and Liu, 2011), antioxidative (Morikawa et al., 2006), sedative and hypnotic (Lin, 2009), immunoregulatory (Li et al., 2006), antiaging (Si and Liu, 2013), and antihyperlipidemic (Zhang et al., 2013) activities. In recent years, tubers of *G. conopsea* have also been used as an ingredient and tonic added to food to strengthen bodies and prevent illness (Shang et al., 2017). Owing to overexploitation, habitat destruction, and its low natural reproductive capacity, the *G. conopsea* populations are shrinking rapidly (Lin et al., 2020; Shang et al., 2017; Gao et al., 2020). *G. conopsea* has been listed as a grade II endangered species by the Convention on International Trade in Endangered Species of Wild Fauna and Flora (CITES) (Lin et al., 2020).

Fungal endophytes are a group of nonpathogenic fungal organisms that can enter plants through the root cortex, wounds, or stomata and reside in different tissues or organs without causing any harm to the host plants, with benefits for both the host and fungus (Tan and Zou, 2001; Schulz and Boyle, 2005; Kusari et al., 2013). Previous studies have indicated that endophytic fungi can be considered the second genome of the plant, playing a crucial role in nutrient cycling and plant growth and development, for example, by conferring resistance to various biotic and abiotic stresses (Seleiman et al., 2021), producing biocontrol agents and phytohormones (Gong et al., 2020; Firáková et al., 2007), secreting extracellular enzymes to promote nutrient cycling (Chathurdevi and Gowrie, 2016), and producing bioactive properties (Omomowo et al., 2023). Owing to the tiny seeds of orchids lacking endosperm and sufficient nutrients, seed germination under natural conditions requires mycorrhizal fungal colonization for nutrient supply (Lin et al., 2020). Previous studies have shown that mycorrhizal fungi can facilitate seed germination, protocorm growth, and seedling establishment in orchids (Wang et al., 2024; Huynh et al., 2009; Bonnardeaux et al., 2007; Sathiyadash et al., 2012). In comparison to soil-inhabiting fungi, dominant endophytic fungal taxa exhibit greater stability, and these may represent key fungal symbionts influencing orchid growth and development (Chen et al., 2019). A culturable orchid mycorrhizal (OM) fungal strain of *Epulorhiza* sp. isolated from the rhizome significantly promoted seed germination in *Dendrobium officinale* and seedling growth in *Epidendrum secundum* orchids (Wang et al., 2024). The mycorrhizal fungus *Ceratobasidium* GS2, isolated from *G. conopsea*, produces several steroid compounds that significantly promote the growth and differentiation of its protocorms (Shi et al., 2023). These findings suggest that symbiotic fungi may play a crucial role in the symbiotic germination of *G*. *conopsea* seeds via metabolite-mediated interactions. In recent years, endophytic fungi have garnered significant attention due to their potential applications in pharmacology, ecology, and agroindustry (Jha et al., 2023). However, the composition and diversity of endophytic fungal communities within different tissues of *G. conopsea* remain largely unexplored, underscoring the necessity of this study.

Microbial community assembly, based on the niche and neutral theories, is a crucial determinant of microbial diversity and ecological function, which are shaped by both deterministic and stochastic processes (Zhang et al., 2024). The deterministic processes are based on the niche theory, which posits that environmental filtering, interspecies interactions and species characteristics govern microbial community structure (Vellend, 2010). The neutral theory emphasizes that stochastic processes, such as gene and ecological drift, birth and death, determine microbial community dynamics (Ning et al., 2019). Many studies have shown that microbial community assembly is influenced by environmental factors, host species and tissue type (Kivlin et al., 2022; Zheng and Gong, 2019; Carper et al., 2018). Moreover, host metabolites, such as salicylic acid, triterpenoids and benzoxazines, also affect community assembly (Pang et al., 2021; Lebeis et al., 2015). However, the process of endophytic fungal community assembly in different tissues of *G. conopsea* remains unclear. In this study, high-throughput sequencing technology was used to investigate the diversity and composition of the endophytic fungal community in different tissues of *G. conopsea*. Subsequently, correlation network analysis, fungal community assembly and functional prediction were conducted to elucidate endophytic fungal community dynamics and function.

## 2 Materials and methods

### 2.1 Sample collection and treatment

In August 2019, the roots, stems, leaves, and fruit of *G. conopsea* plants (5 years old) were collected from Linzhi city, Xizang, China (geographical coordinates: 94°41′51.90^′′^E and 29°37′ 0.85^′′^N), and the altitude of the sampling site ranged from 3,244 m to 4,513 m, with a highland, temperate, semihumid climate, an annual average temperature of −0.73 °C, and an annual average precipitation of 1,134 mm. Fifteen biological replicates were collected for each tissue type, resulting in a total of 60 samples (15 replicates × 4 tissue types). Each replicate was sampled from 45 individual 5-year-old *G. conopsea* plants exhibiting uniform growth and no signs of disease or pest infestation. Each sample will undergo independent sequencing following subsequent processing. GcR represents the tuber samples, GcL represents the leaf samples, GcS represents the stem samples, and GcF represents the fruit samples of *G. conopsea*. The collected samples were randomly packed in sterile plastic bags and brought to the laboratory. Tissue samples were rinsed thoroughly with ultrapure water, followed by surface washing of *G. conopsea* roots, stems, leaves, and fruits with sterile water in a laminar flow hood. Surface moisture was blotted dry using sterile filter paper. Samples were then immersed in 75% ethanol for 30 s, rinsed 2–3 times with sterile water, and subsequently soaked with agitation in 10% sodium hypochlorite solution for 3–5 min. After rinsing 2–3 times with sterile water and drying with sterile filter paper, the samples were stored for further use. If surface sterilization of root and fruit tissues remained insufficient, the entire procedure was repeated up to multiple times until sterility was confirmed. The sterile water from the final rinse was inoculated onto PDA (Potato Dextrose Agar) medium as a control and incubated at 28 °C for 2–3 days. Surface sterilization was deemed complete if no microbial growth was observed.

### 2.2 DNA extraction, PCR amplification, and sequencing

Total DNA was extracted from all the samples using fungal genomic DNA extraction kits (Solarbio, D2300). Afterward, the quality of extracted DNA was assessed by 1% agarose gel electrophoresis, and the concentration and purity were determined with a NanoDrop 2000 UV–vis spectrophotometer (Thermo Scientific, Wilmington, USA). The primers ITS1F(F) (5′-CTTGGTCATTTAGAGGAAGTAA3′) and ITS2(R) (5′-GCTGCGTTCTTCATCGATGC3′) were used to amplify the ITS1 region from all the fungal samples (Chen et al., 2019; Liang et al., 2022), and PCR amplification was performed using a 20 μL reaction system (5 × TransStart FastPfu buffer, 4 μL; 2.5 mM dNTPs, 2 μL; 5 μM primer ITSIF, 0.8 μL; 5 μM primer ITS2R, 0.8 μL; TransStart FastPfu DNA Polymerase, 0.4 μL; template DNA, 10 ng; and ddH_2_O up to 20 μL). The PCR procedure was as follows: initial denaturation at 95 °C for 3 min; 27 cycles of denaturation at 95 °C for 30 s, annealing at 55 °C for 30 s and extension at 72 °C for 45 s; and a single extension at 72 °C for 10 min, followed by holding at 4 °C. The PCR products were detected by 2% agarose gel electrophoresis. An AxyPrep DNA Gel Extraction Kit (Axygen Biosciences, Union City, CA, USA) was used for purification of the products. The purified products were sent to Shanghai Majorbio Biopharm Technology Co., Ltd., for sequencing, and the sequences were deposited into the NCBI Sequence Read Archive (SRA) database (accession number: PRJNA699676).

### 2.3 Statistical analysis

The raw gene sequencing reads were quality filtered and merged by FLASH (Magoc and Salzberg, 2011). Operational taxonomic units (OTUs) with a 97% similarity cutoff were clustered using UPARSE (version 7.0.1), and chimeric sequences were removed (Edgar, 2013). The taxonomy of each OTU representative sequence was analyzed by RDP Classifier (version 2.2) (Zhao et al., 2018) against the UNITE database (version 8.0) (Kõljalg et al., 2013) with a confidence threshold of 70%. Alpha diversity indices, including the Sobs, Chao, Shannon, and coverage indices, were determined for all the samples using Mothur (version 1.30.2) to analyze the richness, diversity, and coverage of the microbial community. A Venn diagram, which could intuitively show the composition similarities and overlap of species, was used to analyze the number of common and unique OTU in multiple groups (Wang et al., 2019). Analysis of fungal community composition among all samples was performed with the R package at different taxonomic levels. Principal coordinate analysis (PCoA) was implemented with QIIME (v1.9.1) (Yuan et al., 2018). The functions of the endophytic fungi were predicted using the FUNGuild database (Nguyen et al., 2016). Cooccurrence network analysis was conducted on the basis of Spearman's correlation to explore the interactions among fungal genera with absolute correlation coefficients ≥0.7 and *p* < 0.05, and the results were visualized using Gephi (0.9.7) (Chen et al., 2022). A neutral community model (NCM) was used to assess the microbial community assembly process (Li et al., 2024). Differences were analyzed using Duncan's multiple comparison tests and one-way analysis of variance (ANOVA) by SPSS 26.0 software at a significance level of 0.05.

## 3 Results

### 3.1 Diversity of endophytic fungi in different tissues

**Figure 1:**
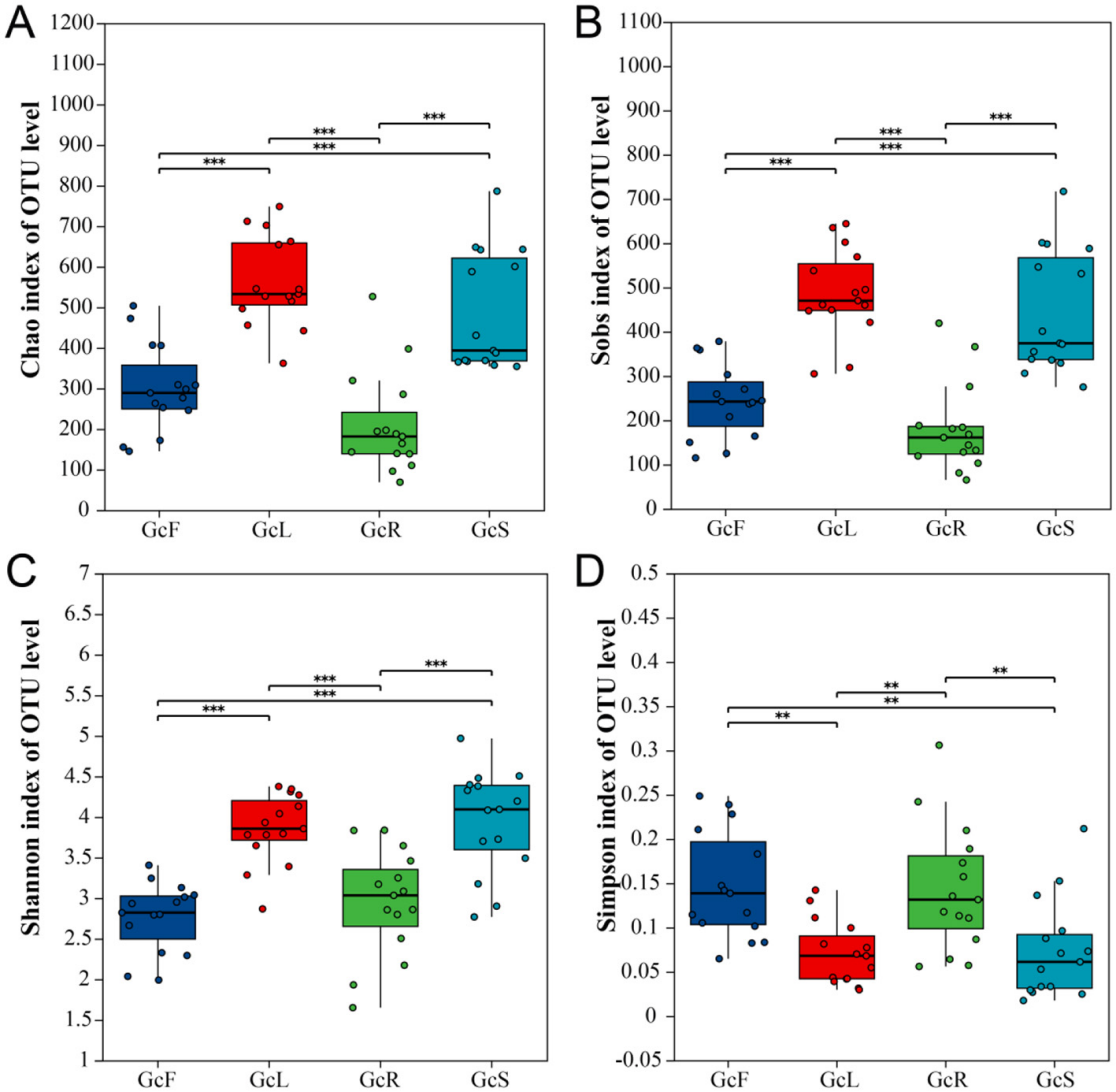
Alpha diversity analysis in different tissues of *G. conopsea*. **(A)** Chao1 index; **(B)** Sobs index; **(C)** Shannon index; **(D)** Simpson index. GcR represents root tissues, GcL represents leaf tissues, GcS represents stem tissues, GcF represents fruit tissues. Significance levels were presented as follows: **P < 0.01 and ***P < 0.001.

After filtering the sequencing data, a total of 249,093 valid sequences (with numbers ranging from 57,304 to 64,605 valid sequences in each sample) were obtained from 60 samples, with an average length of 241 bp. The fungal dataset yielded 7,371 OTUs. Among them, the number of OTUs in GcL was highest, whereas that in GcR was lowest, the lowest number of OTUs in root samples may be due to the fact that some dominant mycorrhizal fungi in the roots of photosynthetic orchids were not amplified due to mismatches with the primers used (Table 1). The rarefaction curves, combined with the coverage index analysis results, indicated that the sequencing depth was appropriate, and the sequencing results reflected the endophytic fungal diversity in all the samples (Supplementary Figure S1, Table 1). The alpha diversity analysis revealed significant differences in the Chao1 and Sobs indices among the different tissues (*P* < 0.05), the richness of GcL and GcS was highest, and that of GcR was lowest; Similarly, significant differences in Shannon and Simpson indices were observed among different tissues (*P* < 0.05) (Figure 1).

**Table 1:** Sample sequencing data statistics.

**Sample**	**Sequence number**	**Bases number (bp)**	**Average length (bp)**	**Coverage (%)**	**OTUs**
GcR	57,304	14,564,954	256	99.91	1,243
GcS	64,605	15,473,110	240	99.84	2,331
GcL	64,347	15,028,188	234	99.73	2,407
GcF	62,837	14,604,344	233	99.82	1,390

GcR represents root tissues, GcL represents leaf tissues, GcS represents stem tissues, GcF represents fruit tissues.

**Figure 2:**
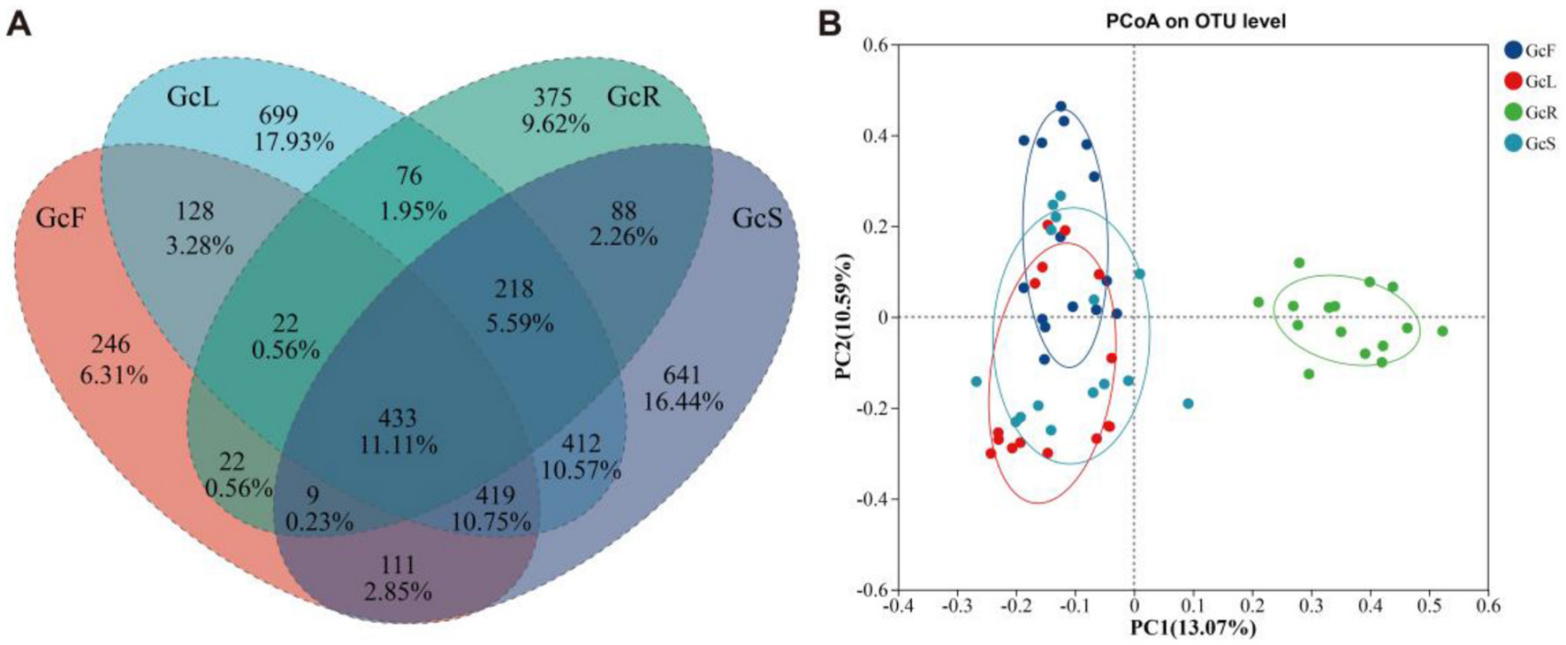
Venn diagram and PCoA analysis of endophytic fungal community among different tissues of *G. conopsea*. **(A)** Venn diagram; **(B)** PCoA analysis. GcR represents root tissues, GcL represents leaf tissues, GcS represents stem tissues, GcF represents fruit tissues.

According to the OTU cluster analysis, there were 433 shared OTUs among the different tissues of *G. conopsea*, accounting for 11.11%, and the percentages of unique OTUs of GcR, GcL, GcS, and GcF were 375 (9.62%), 699 (17.93%), 641 (16.44%), and 246 (6.31%), respectively (Figure 2A). To explore the effects of different tissues on the endophytic fungal community, PCoA was performed at the OTU level on the basis of the Bray–Curtis distance. The results suggested that the distance between root tissues and other tissues of *G. conopsea* was relatively large, and the different contribution rates of PC1 and PC2 were 13.07 and 10.59%, respectively, suggesting that the endophytic fungal community in root tissues significantly differed from that in other tissues (Figure 2B). The samples from stem, leaf, and fruit tissues were relatively clustered, suggesting that the endophytic fungal communities were similar.

### 3.2 Analysis of endophytic fungal community composition

**Figure 3:**
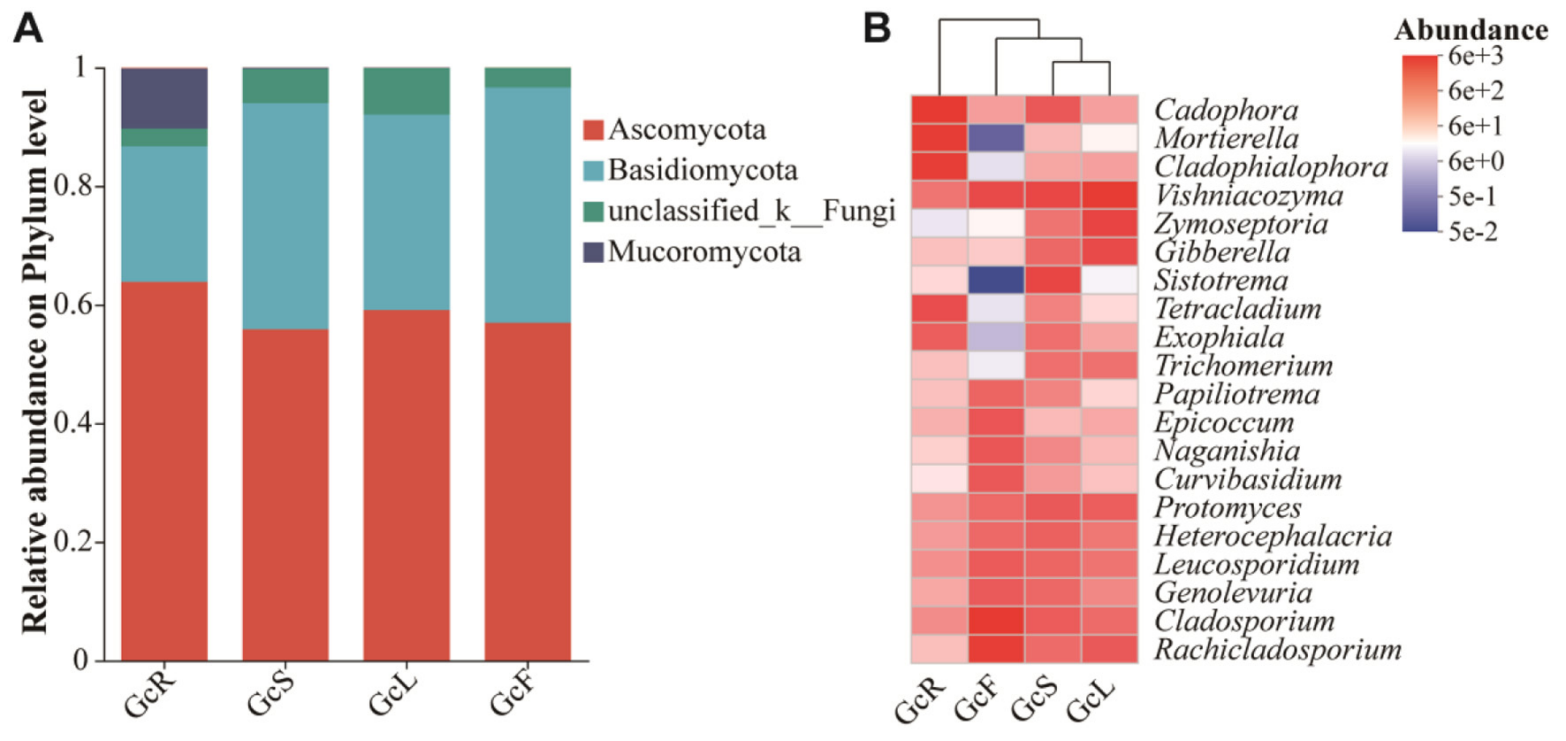
Relative abundance of endophytic fungi in different tissues of *G. conopsea* at the phylum level **(A)** and the genus level **(B)**. GcR represents root tissues, GcL represents leaf tissues, GcS represents stem tissues, GcF represents fruit tissues.

The ITS sequences obtained from 60 tissue samples were classified into 12 phyla, 37 classes, 113 orders, 294 families, and 621 genera at the 97% similarity level. The endophytic fungal community composition and relative abundance varied among different tissues of *G. conopsea* (Supplementary Table S1, Figure 3). As shown in Figure 3A, the dominant fungal phyla (relative abundance >1%) were Ascomycota, Basidiomycota, and Mucoromycota across all the samples. Ascomycota was the dominant phylum, with a relative abundance ranging from 56.96% to 63.83%, followed by Basidiomycota, with a relative abundance ranging from 22.87% to 39.67%, whereas the relative abundance of Mucoromycota in root tissues was greater than that in other tissues, at 10.13%, and that in leaf, stem, and fruit tissues was less than 1%. The composition of the endophytic fungal community at the genus level and clustering of the top 20 genera in terms of relative abundance were shown in Supplementary Figure S2 and Figure 3B. The distributions of the dominant genera significantly differed among the different tissues. In the GcR samples, the dominant genera (relative abundance >1%) were *Cadophora* (16.45%), *Mortierella* (10.08%), *Cladophialophora* (9.41%), *Tetracladium* (4.03%), *Ceratobasidium* (2.63%), *Russula* (2.52%), *Exophiala* (2.30%), *Hyaloscypha* (2.01%), *Trichocladium* (1.02%), *Vishniacozyma* (1.13%), *Ilyonectria* (1.93%), and *Phialocephala* (1.20%). In the GcS samples, the dominant genera (relative abundance >1%) were *Sistotrema* (5.41%), *Vishniacozyma* (5.09%), *Cadophora* (2.78%), *Protomyces* (2.69%), *Cladosporium* (2.48%), *Heterocephalacria* (2.10%), *Leucosporidium* (1.81%), *Gibberella* (1.71%), *Genolevuria* (1.71%), *Rachicladosporium* (1.49%), *Exophiala* (1.37%), *Trichomerium* (1.37%), *Knufia* (1.24%), *Zymoseptoria* (1.15%), *Plenodomus* (1.14%), *Mycocentrospora* (1.09%), and *Filobasidium* (1.00%). In the GcL samples, the dominant genera (relative abundance >1%) were *Vishniacozyma* (12.31%), *Zymoseptoria* (5.93%), *Gibberella* (4.46%), *Rachicladosporium* (2.69%), *Protomyces* (2.29%), *Podospora* (1.84%), *Ramularia* (1.56%), *Cladosporium* (1.54%), *Trichomerium* (1.28%), *Leucosporidium* (1.17%), *Knufia* (1.13%), *Heterocephalacria* (1.06%), and *Septoria* (1.01%). In the GcF samples, the dominant genera (relative abundance >1%) were *Cladosporium* (16.17%), *Rachicladosporium* (9.20%), *Vishniacozyma* (4.39%), *Epicoccum* (3.08%), *Leucosporidium* (2.58%), *Genolevuria* (2.59%), *Naganishia* (2.97%), *Curvibasidium* (2.82%), *Papiliotrema* (1.87%), *Heterocephalacria* (1.64%), *Protomyces* (1.59%), and *Plenodomus* (1.03%). The clustering of dominant genera analysis shown that the composition of endophytic fungal community was similar between stem and leaf tissues, and varied between roots and the other two tissues.

### 3.3 Genus-level differential analysis across distinct tissue types

**Figure 4:**
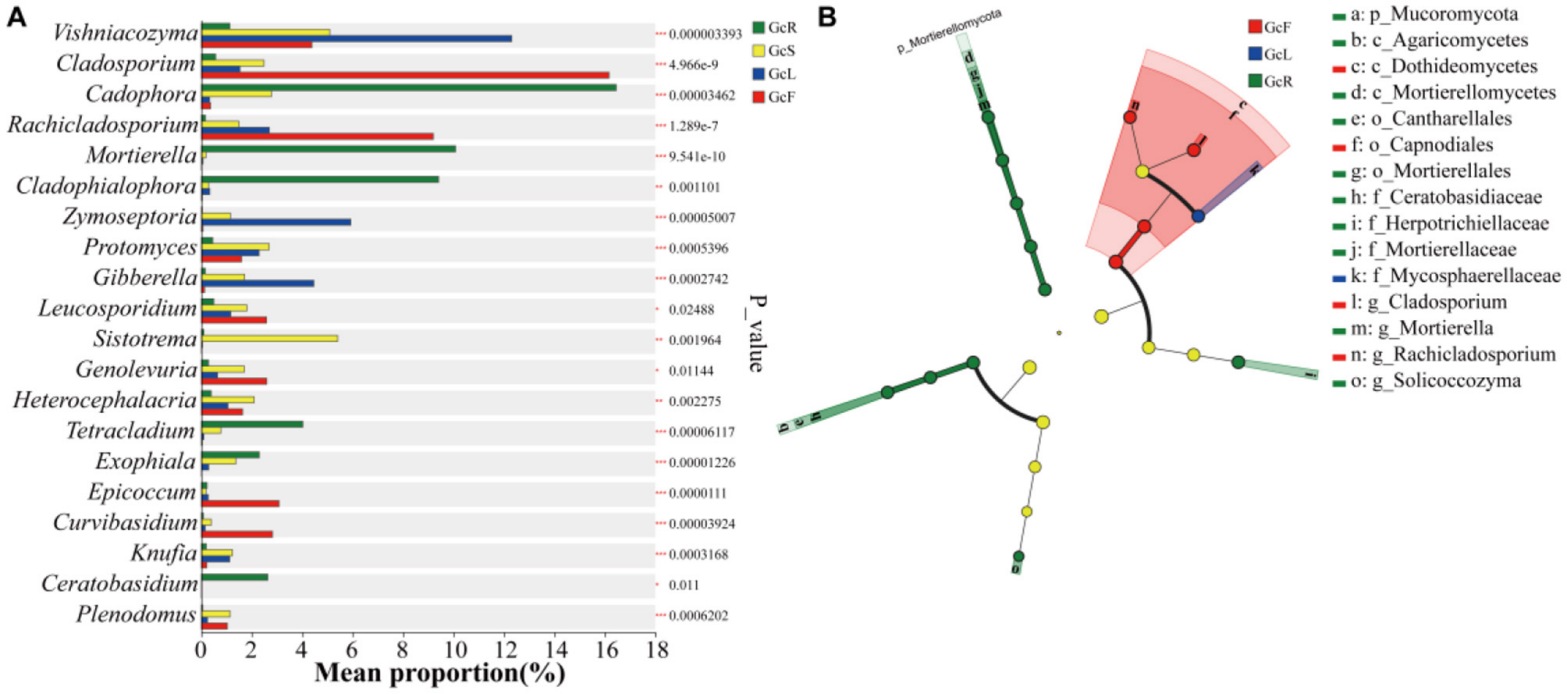
Statistical comparison of relative abundance based on the Kruskal-Wallis rank sum test **(A)** and LEfSe differential analysis **(B)** among different tissues of *G. conopsea*. The phylogenetic tree diagram illustrates taxonomic differences across hierarchical levels, providing a visual representation of differentially abundant taxa identified between groups at various taxonomic ranks. Nodes with distinct colors indicate microbial taxa that are significantly enriched in corresponding groups and exert a significant impact on inter-group differences; pale yellow nodes represent taxa with no significant differential abundance across groups or no substantial contribution to inter-group distinctions. When the number of significantly differential taxa is ≤ 50, the legend on the right is displayed in a single column; when >50, the legend on the right is presented in two columns.

The distribution of the top 20 genera in different tissues were compared (Figure 4A). The relative abundances of *Cadophora, Mortierella, Cladophialophora, Tetracladium, Exophiala*, and *Ceratobasidium* were significantly greater in root tissues than in other tissues (*p* < 0.05). The relative abundances of *Protomyces, Sistotrema, Heterocephalacria, Knufia*, and *Plenodomus* were significantly greater in stem tissues than in other tissues (*p* < 0.05). The relative abundances of *Vishniacozyma, Zymoseptoria*, and *Gibberella* were significantly greater in the leaf tissues than in other tissues (*p* < 0.05). The relative abundances of *Cladosporium, Rachicladosporium, Leucosporidium, Genolevuria, Epicoccum*, and *Curvibasidium* were significantly greater in fruit tissues than in other tissues (*p* < 0.05). Overall, the relative abundance of endophytic fungi significantly differed among different tissues.

Linear discriminant analysis Effect Size (LEfSe) enables multi-level taxonomic differential analysis (spanning phylum, class, order, family, genus, and species ranks) to test for differential taxa across multiple hierarchical levels. It quantifies the impact of each taxon on the observed differences using the Linear Discriminant Analysis (LDA) score, indicating potential key roles of these taxa in response to environmental changes. LEfSe (Figure 4B) revealed 15 fungal biomarkers at the phylum, class, order, family and genus levels on the basis of the criterion LDA>4. Among these, p_Mucoromycota, c_Mortierellomycetes, c_Agaricomycetes, o_Cantharellales, o_Mortierellales, f_Ceratobasidiaceae, f_Herpotrichiellaceae, g_*Mortierella*, and g_*Solicoccozyma* were identified in the root tissues; c_Dothideomycetes, o_Capnodiales, g_*Cladosporium*, and g_*Cladosporium* were identified in the fruit tissues; and f_Mycosphaerellaceae was identified in the leaf tissues. However, no fungal biomarkers were detected in the stem tissues.

### 3.4 Cooccurrence network analysis of the endophytic fungal community in different tissues

**Figure 5:**
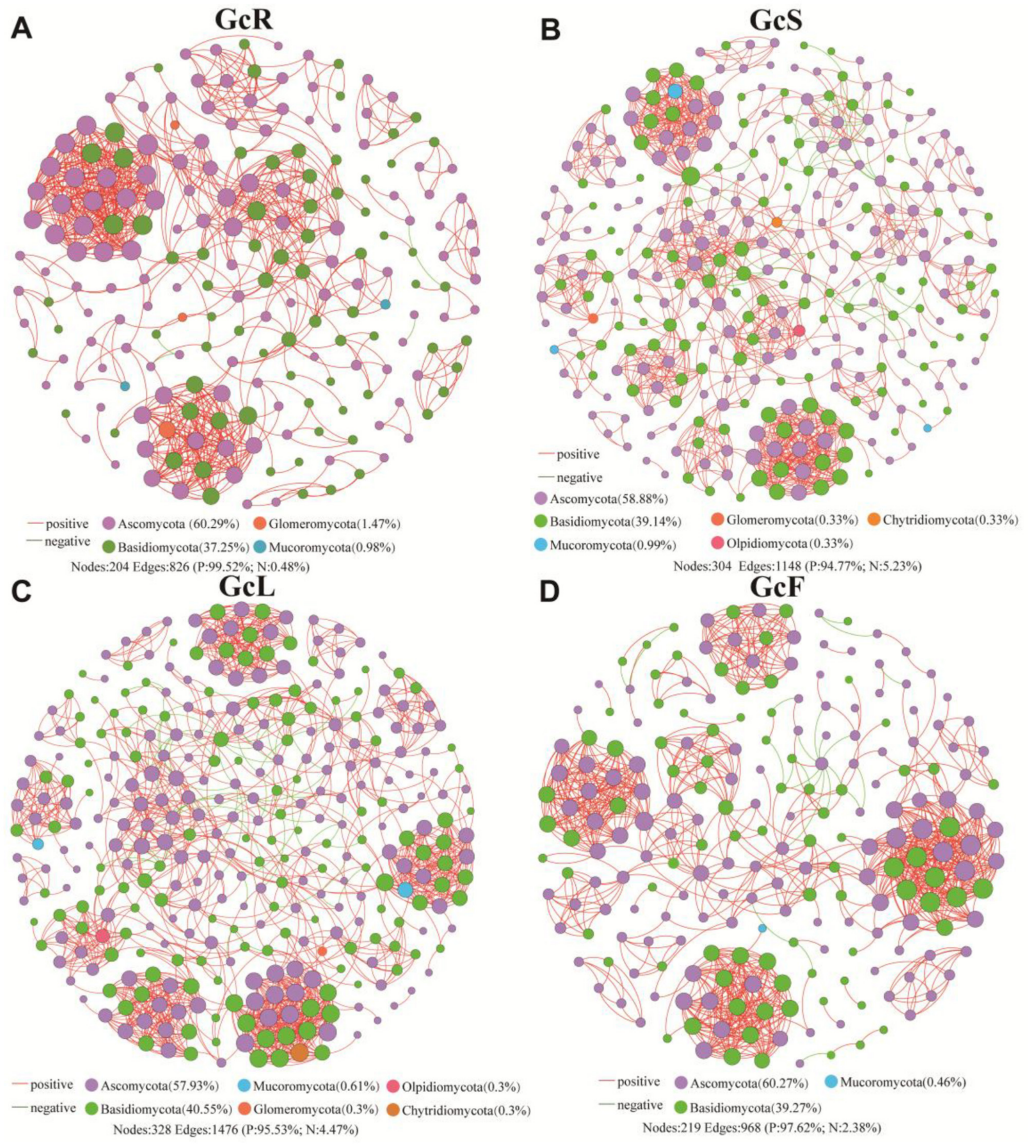
Co-occurrence network analysis of endophytic fungi in different tissues. **(A)** root tissues, **(B)** stem tissues, **(C)** leaf tissues, **(D)** fruit tissues.

To explore the potential interactions within endophytic fungi among different tissues, co-occurrence network analysis was performed at the genus level (Figure 5). The correlation network analysis revealed that the positive proportions were greater than the negative proportions in the GcR, GcS, GcL, and GcF tissues; the positive proportions ranged from 94.77% to 99.52%. In the GcR tissues, a total of 204 nodes and 826 edges were significantly correlated (*P* < 0.05, |rho| > 0.7), with an average degree of 8.098 and clustering coefficient of 0.677; the nodes were mainly affiliated with Ascomycota (60.29%), Basidiomycota (37.25%), Glomeromycota (1.47%), and Mucoromycota (0.98%). In the GcS tissues, a total of 304 nodes and 1,148 edges were significantly correlated (*P* < 0.05, |rho| > 0.7), with an average degree of 7.553 and clustering coefficient of 0.620; the nodes were mainly affiliated with Ascomycota (58.88%), Basidiomycota (39.14%), Mucoromycota (0.99%), Glomeromycota (0.33%), Chytridiomycota (0.33%), and Olpidiomycota (0.33%). In the GcL tissues, a total of 328 nodes and 1,476 edges were significantly correlated (*P* < 0.05, |rho| > 0.7), with an average degree of 9.000 and clustering coefficient of 0.637; the nodes were mainly affiliated with Ascomycota (57.93%), Basidiomycota (40.55%), Mucoromycota (0.61%), Glomeromycota (0.3%), Olpidiomycota (0.3%), Chytridiomycota (0.3%). In the GcF tissues, a total of 219 nodes and 968 edges were significantly correlated (*P* < 0.05, |rho| > 0.7), with an average degree of 8.440 and clustering coefficient of 0.712; the nodes were mainly affiliated with Ascomycota (60.27%), Basidiomycota (39.27%), and Mucoromycota (0.46%). The results revealed that the complexity of endophytic fungi significantly differed among different tissues.

### 3.5 Assembly processes of the endophytic fungal community

**Figure 6:**
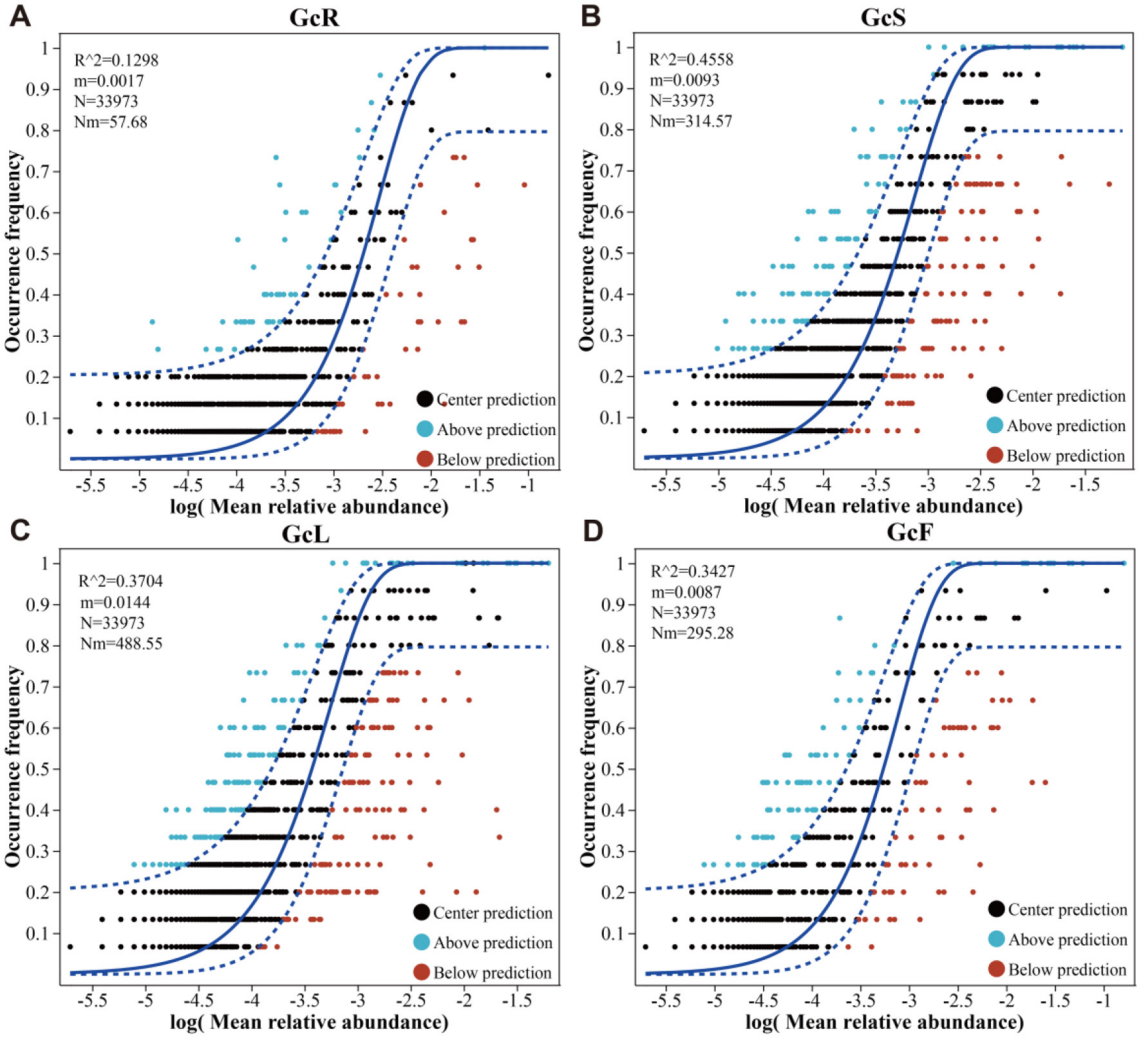
Endophytic fungal community assembly in different tissues of *G. conopsea*. **(A)** root tissues, **(B)** stem tissues, **(C)** leaf tissues, **(D)** fruit tissues.

NCM-based analysis revealed distinct assembly dynamics of endophytic fungal communities across different tissues (Figure 6). The goodness of fit (*R*^2^) for the endophytic fungal communities in the GcS sample (*R*^2^ = 0.4458) was greater than that in the GcR sample (*R*^2^ = 0.1298), GcL sample (*R*^2^ = 0.3704), and GcF sample (*R*^2^ = 0.3427), showing an initial rapid increase followed by a gradual decrease in the explanatory rate for *G. conopsea* from root tissues to fruit tissues. Therefore, the endophytic fungal community assembly in root tissues was more strongly influenced by deterministic processes, and the deterministic processes in the GcS, GcL, and GcF samples became less important. These results indicated that the degree of diffusion for endophytic fungal communities in the GcR sample was limited, while that in other tissues gradually increased.

### 3.6 Functional prediction for endophytic fungi in different tissues

**Figure 7:**
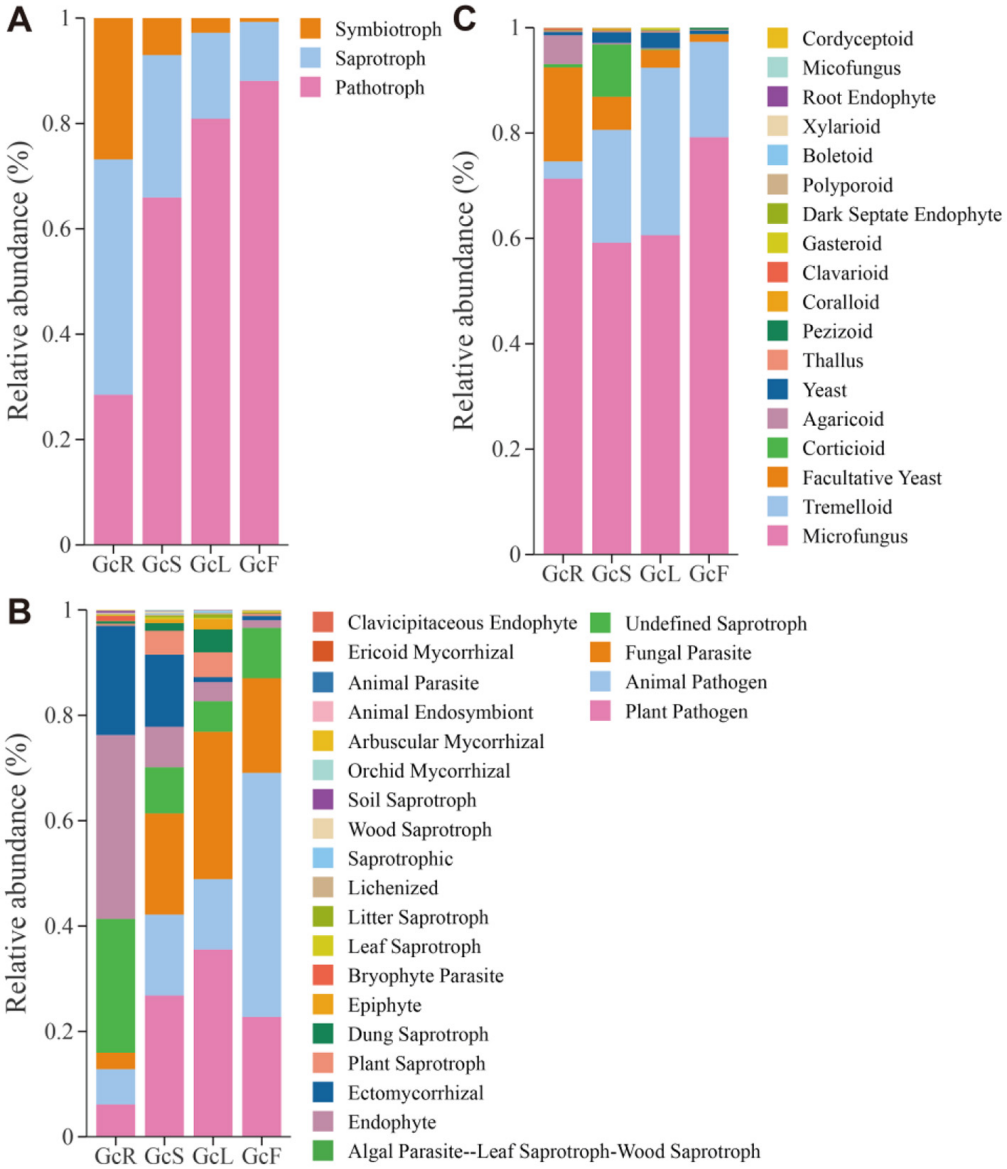
Functional prediction of endophytic fungi in different tissues of *G. conopsea*. **(A)** At the trophic mode level. **(B)** At the guilds level. **(C)** At the growth morphology level. GcR, root tissues, GcL, leaf tissues, GcS, stem tissues, GcF, fruit tissues.

The FUNGuild database was used to predict the potential functions of endophytic fungi in different tissues of *G. conopsea* on the basis of ecological guilds. Fungal OTUs were classified into three ecologically relevant trophic modes (pathotrophs, saprotrophs, and symbiotrophs). Of these, the relative abundance of pathotrophs (28.5%−88.1%) gradually increased from the root to fruit tissues, while that of saprotrophs (44.7%−11.2%) and symbiotrophs (26.8%−0.7%) gradually decreased from root to fruit tissues (Figure 7A). At the guild level, endophytes (34.9%), undefined saprotrophs (25.4%), and ectomycorrhizal fungi (20.7%) were the top three high abundance guilds in the root tissues; plant pathogens (26.8%), fungal parasites (19.2%), animal pathogens (15.4%), and ectomycorrhizal fungi (13.7%) were the top four high-abundance guilds in the stem tissues; and plant pathogens (35.5%), fungal parasites (28.0%), and animal pathogens (13.4%) were the top three high-abundance guilds in the leaf tissues. Animal pathogens (46.3%), plant pathogens (22.8%), and fungal parasites (18.0%) were the top three high-abundance guilds in the fruit tissues (Figure 7B). The growth morphology in the root tissues was associated with microfungi (71.3%) and facultative yeasts (17.8%), whereas that in the stem tissues was associated with microfungi (59.2%), tremelloid fungi (21.4%), and corticioid fungi (9.9%), and that in the leaf and fruit tissues was associated with microfungi (60.6 and 79.2%) and tremelloid fungi (31.8 and 18.1%) (Figure 7C). These results indicated that the relative abundance of endophytic fungi in different tissues significantly differed at the three functional levels.

## 4 Discussion

Extensive studies on endophytic plant interactions have focused mainly on endophytic microbes because of their potential functions in pharmaceuticals, ecology, industry and agriculture (Jha et al., 2023). In this study, we explored the endophytic fungal community structure and assembly in different tissues of *G. conopsea*. Prior to conducting relevant analyses, the sample sequences were first rarefied according to the minimum number of sequences per sample. The present study revealed that the endophytic fungal communities in the GcL and GcS samples presented the greatest number of OTUs, followed by those in the GcF and GcR samples. The richness indices of the GcS and GcL samples were significantly greater than those of the GcF and GcR samples. The richness index of the GcR samples was the lowest; however, there were no significant differences in the diversity indices among the different tissues. Previous studies have shown that endophytes have unique tissue specificity and are affected by various factors, such as climate, geographic location, genotype, growth stage, seasonality, and metabolites (Zhang et al., 2023; Yang et al., 2023; Li et al., 2025; Wang et al., 2022). Saha et al. reported that the diversity of endophytic fungi in *Chromolaena odorata* was highest in stems, followed by roots and leaves (Saha, 2024). Pooja et al. (2025) reported that the diversity in *Lagenandra toxicaria* root samples was greater than that in leaf samples. These results indicated that the endophytic diversity pattern was not universal.

The endophytic fungi in different tissues of *G. conopsea* were classified into 12 phyla, 37 classes, 113 orders, 294 families, and 621 genera. Ascomycota, Basidiomycota, and Mucoromycota were the dominant phyla; however, the relative abundance of Mucoromycota in root tissues, at 10.13%, was greater than that in other tissues, at less than 1% in leaf, stem, and fruit tissues. These findings corroborate previous studies showing that Ascomycota, Basidiomycota, and Mucoromycota were the predominant fungal phyla in the root tissues of *G. conopsea* (Lin et al., 2020). Previous studies have shown that endophytic fungi from different tissues belong to Ascomycota, Basidiomycota, and Zoopagomycota (Saha, 2024). Some species of Ascomycota and Basidiomycota participate in the carbon cycle by degrading organic matter (Unterseher et al., 2013), whereas Mucoromycota species have various agricultural benefits (Li et al., 2020), and the relative abundance of Mucoromycota is related to altitude and soil substrate (SOM, N, P, and K) (Ren et al., 2024). At the genus level, the dominant endophytic fungal community in different tissues differed, which might be related to tissue type and metabolites that varied among different tissues (Li et al., 2025; Wang et al., 2022). The germination of Orchidaceae seeds depends on mycorrhizal fungal colonization under natural conditions (Li et al., 2020). Previous studies have shown that endophytic fungi, such as *Gibberella* (Silva et al., 2018), *Leptosphaerulina* (Favre-Godal et al., 2020), *Trichoderma* (Zhang et al., 2016), *Cladosporium* (Favre-Godal et al., 2020), and *Cadophora* (Berthelot et al., 2016) species, can produce phytohormones. The mycorrhizal fungus *Ceratobasidium* GS2, isolated from *G. conopsea*, produces several steroid compounds that significantly promote the growth and differentiation of its protocorms (Shi et al., 2023). This study revealed that the relative abundances of *Ceratobasidium, Cadophora*, and *Mortierella* were significantly higher in root tissues than in other tissues. Furthermore, *Ceratobasidium* is classified under Basidiomycota, which was the dominant phylum across the four tissue types. These results indicate that roots harbor a relatively high abundance of growth-promoting fungi, suggesting that *G. conopsea* roots should be prioritized as the material for isolating growth-promoting endophytic fungi. In the GcR samples, the dominant genus *Ceratobasidium* had a relative abundance of 2.63%, which is relatively low for a genus composed of mycorrhizal fungi. We hypothesize two potential explanations for this phenomenon: First, the low abundance of *Ceratobasidium* may be one of the factors contributing to the endangered status of *G*. *conopsea*, though this remains merely a preliminary speculation. Second, other dominant mycorrhizal fungi may have colonized the roots of *G*. *conopsea* but were not amplified by the ITS primers used in this study. This speculation could be further validated through microscopic observation of the mycorrhizal morphology in *G*. *conopsea* roots in subsequent research. *Mortierella* species have potential functions in biocontrol, phosphorus absorption, and phytohormone production (Yakun, 2024). *Cadophora* (16.45%) and *Mortierella* (10.08%) were significantly enriched in the root tissues of *G. conopsea, Cladosporium* (1.54%−16.17%) was the dominant genus in the stem, leaf, and fruit tissues, and *Gibberella* was significantly enriched in the stem and leaf tissues. These genera might play crucial roles in the growth of *G. conopsea*.

A co-occurrence network not only can reveal more microbial complex interactions among different microbial taxa but is also a useful tool for identifying the key species that play crucial roles in maintaining microbial community stability (Xu et al., 2024). These highly complex microbial networks contribute to resisting biotic and abiotic stress to maintain host health (Qiao et al., 2024), which can be reflected by the number of edges, nodes, modularity index, and other topological parameters (Fan et al., 2022). In this study, the numbers of nodes and edges in the leaf (328, 1476) and stem (304, 1148) tissues were greater than those in the fruit (219, 968) and root (204, 804) tissues, and the modularity indices of the different tissues were greater than 0.4, indicating a stable modular structure. The smallest number of nodes and edges in root samples may be attributed to the failure of amplification of some dominant mycorrhizal fungi in the roots of *G. conopsea* due to mismatching with the primers used. The results corroborated those of a previous study showing that the endophytic fungal co-occurrence network was more complex in the leaf tissues than in the root tissues of medicinal plants (He et al., 2023). Moreover, these indices suggested that the endophytic fungal community in various tissues of *G. conopsea* exhibited complex network relationships. The *G. conopsea* samples were collected from high-altitude areas that were subject to multiple environmental pressures, such as intense UV-B radiation, low temperatures, and strong winds. Endophytic fungi associated with *G. conopsea* may assist their host in resisting abiotic stresses through highly intricate interactions, thereby endeavoring to maintain host health. In addition, the network analysis revealed that the network structure was mainly positive (≥94.77%). This result was similar to that of previous studies showing that the endophytic fungal co-occurrence network in *Sophora alopecuroides* tissues was mainly positive (96.6%) (Ju et al., 2024).

Microbial community assembly processes play crucial roles in shaping microbial diversity, composition, and ecological function (Zhang et al., 2024). In our study, we found that deterministic processes play crucial roles in the endophytic fungal community of *G. conopsea*. The endophyte community assembly in *Eucommia ulmoides* (Tang et al., 2024) and *Rhododendron* (Zhang et al., 2024) was mainly affected by stochastic processes, whereas the assembly processes of the root endophytic community in alpine grasslands shift under different climate and precipitation conditions (Wei et al., 2024). In addition, He et al. revealed that endophytic fungal community assembly is more strongly affected by the host than by the season (He et al., 2023). These results indicate that microbial community assembly processes are influenced by multiple biotic and abiotic factors; therefore, species identities, tissue niches and environmental factors might determine endophytic fungal community assembly processes in various tissues of *G. conopsea*. In this study, FUNGuild analysis revealed that the abundances of saprotrophs (44.7%−11.2%) and symbiotrophs (26.8%−0.7%) gradually decreased from root to fruit tissues. The function of saprotrophic fungi is mainly to degrade plant cells (non-host plant cells) to provide essential nutrients for the host, and research has shown that Ascomycota members constitute a large group of saprophytic species that promote nutrient cycling (He et al., 2023). Currently, we lack experimental evidence to support this hypothesis, as this study did not investigate the structure and diversity of the fungal community in the rhizospheric soil and bulk soil of *G. conopsea*. Therefore, additional subsequent experimental evidence is required to verify this hypothesis. Mucoromycota is a widely prevalent fungal phylum in nature, and its members are important lower saprophytic fungi, such as *Mortierella* (Yakun, 2024; Zhou et al., 2024); therefore, the relatively high abundance of Ascomycota and Mucoromycota in the root tissues of *G. conopsea* might be associated with nutrient cycling.

## 5 Conclusions

The various tissues of *G. conopsea* were rich in endophytic fungi, dominated by Ascomycota and Basidiomycota. The richness indices of various tissues were significantly different; however, the diversity was not significantly different, and that in the root tissues being relatively low. The compositions of the endophytic fungal communities in leaf, stem, and fruit tissues were similar, while that in root tissues differed from those in other tissues. The relative abundances of *Ceratobasidium, Cadophora*, and *Mortierella* were significantly higher in root tissues than in other tissues, *G. conopsea* roots should be prioritized as the material for isolating growth-promoting endophytic fungi. Co-occurrence network analysis revealed that endophytic fungi in different tissues presented typical modular structures and were mainly positive. Deterministic processes play crucial roles in the endophytic fungal community of *G. conopsea*. The proportions of pathotrophs, saprotrophs, and symbiotrophs were different among various tissues, and the proportion of pathotrophs was greater than those of saprotrophs and symbiotrophs. Overall, tissue type affects the composition and function of endophytic fungi.

## Data Availability

The datasets presented in this study are publicly available. This data can be found here: https://www.ncbi.nlm.nih.gov/sra, accession number PRJNA699676.
