# Supplementary material for: Exploring the fungal community structure and assembly in different tissues of Gymnadenia conopsea
Source: Front Microbiol. 2025 Oct 10;16:1640133. doi: 10.3389/fmicb.2025.1640133 (PMC12550528; doi:10.3389/fmicb.2025.1640133)
Supplement: Supplementary file 1 [file Supplementary_file_1.docx]

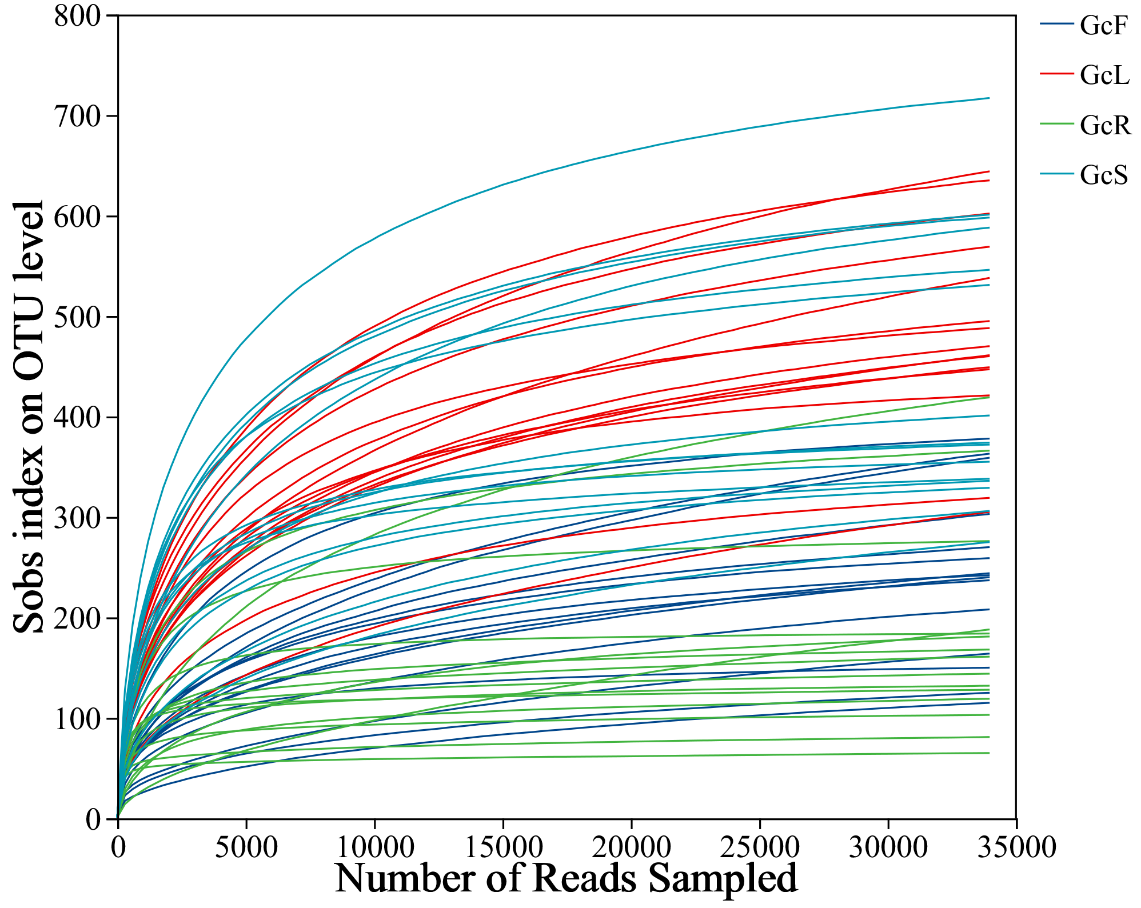


Fig.S1 Rarefaction curves of 60 samples. GcR represents root tissues, GcL represents leaf tissues, GcS represents stem tissues, GcF represents fruit tissues

Table S1 Classified analysis of endophytic fungal taxa detected in different tissues of *G. conopsea.*

| Sample | Phylum | Class | order | family | Genus |
| --- | --- | --- | --- | --- | --- |
| GcR | 8 | 29 | 84 | 190 | 330 |
| GcS | 11 | 30 | 97 | 246 | 458 |
| GcL | 10 | 31 | 93 | 243 | 483 |
| GcF | 5 | 22 | 71 | 181 | 329 |

GcR represents root tissues, GcL represents leaf tissues, GcS represents stem tissues, GcF represents fruit tissues.


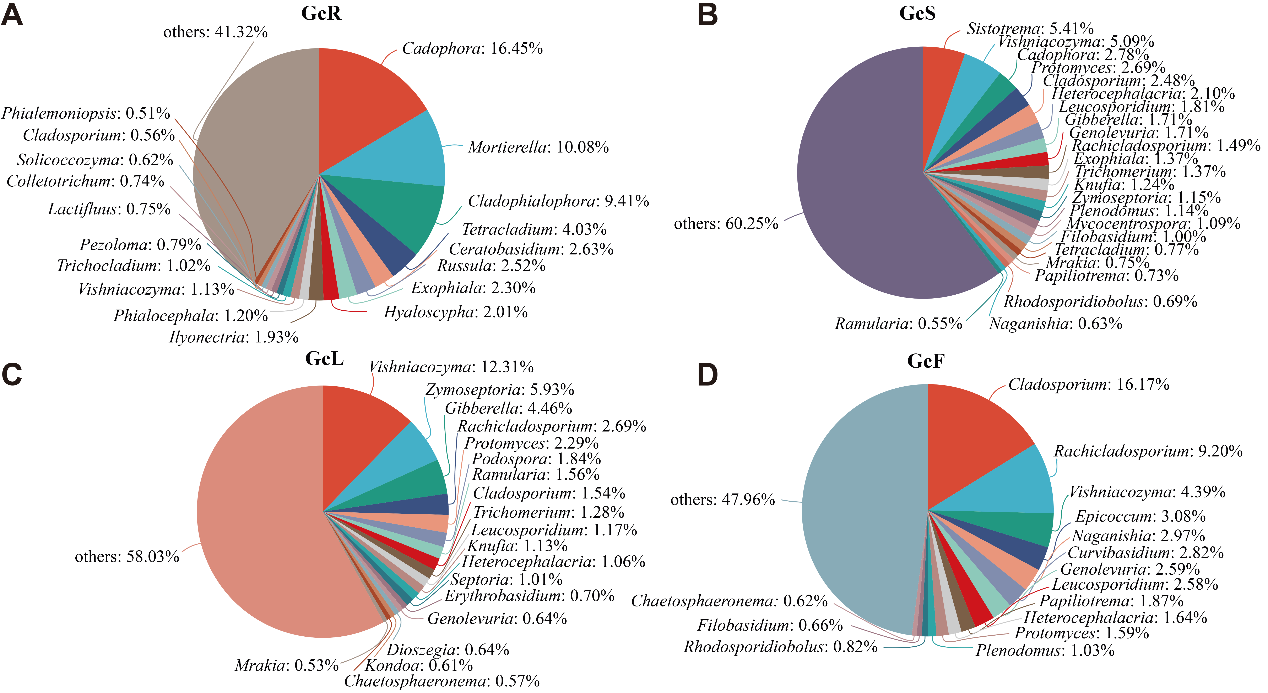


Fig.2S Composition of endophytic fungal community in different tissues of *G. conopsea* at genus level. (A) Composition of endophytic fungal community in root tissues, (B) Composition of endophytic fungal community in stem tissues, (C) Composition of endophytic fungal community in leaf tissues, (D) Composition of endophytic fungal community in fruit tissues.
